# Are Hyoid Bone and Tongue the Risk Factors Contributing to Postoperative Relapse for Mandibular Prognathism?

**DOI:** 10.1155/2016/5284248

**Published:** 2016-03-02

**Authors:** Yu-Chuan Tseng, Steven Lai, Huey-Er Lee, Ker-Kong Chen, Chun-Ming Chen

**Affiliations:** ^1^Graduate Institute of Dental Sciences, College of Dental Medicine, Kaohsiung Medical University, No. 100, Shih-Chuan 1st Road, Kaohsiung 807, Taiwan; ^2^Department of Orthodontics, Kaohsiung Medical University Hospital, No. 100, Shih-Chuan 1st Road, Kaohsiung 807, Taiwan; ^3^Department of Oral and Maxillofacial Surgery, Kaohsiung Medical University Hospital, No. 100, Shih-Chuan 1st Road, Kaohsiung 807, Taiwan

## Abstract

*Objective*. The purpose of this study was to investigate postoperative stability and the correlation between hyoid, tongue, and mandible position following surgery for mandibular prognathism.* Materials and Methods*. Thirty-seven patients, treated for mandibular prognathism using intraoral vertical ramus osteotomy (IVRO), were evaluated cephalometrically. A set of four standardized lateral cephalograms were obtained from each subject preoperatively (T1), immediately postoperatively (T2), six weeks to three months postoperatively (T3), and more than one year postoperatively (T4). The Student *t*-tests, the Pearson correlation coefficient, and the multiple linear regression were used for statistical analysis.* Results*. Immediately after surgery, menton (Me) setback was 12.8 mm, hyoid (H) setback was 4.9 mm, and vallecula epiglottica (V) setback was 5.8 mm. The postoperative stability significantly correlated (*r* = −0.512, *p* < 0.01) with the amount of setback. The hyoid bone and tongue did not have significant effects on postoperative stability. Multiple linear regression model (*R* ^2^ = 0.2658, *p* < 0.05) showed predictability: Horizontal Relapse Me (T4-T2) = −6.406 − 0.488Me (T2-T1) + 0.069H (T2-T1) − 0.0619V (T2-T1).* Conclusion*. Mandibular setback surgery may push the hyoid and tongue significantly backward, but this did not correlate with mandibular relapse. Postoperative stability significantly correlated with the amount of mandibular setback.

## 1. Introduction

The hyoid bone is a horseshoe-shaped bone located in the neck, between the mandible and the thyroid cartilage. Muscles and ligaments are attached to the greater and lesser cornu to connect them to the floor of the mouth, the tongue, the epiglottis, the pharynx, the larynx, the mandible, and the styloid process of the temporal bone [1–3]. The resistance generated between these muscles and the elastic membrane of the larynx and trachea determines the position of the hyoid bone [1, 3, 4]. Adamidis [5] compared the hyoid bone positions of patients with normal occlusion (Angle class I) and patients with Angle class III occlusion and determined that the hyoid bones of patients with Angle class III occlusion were farther forward than were those of patients with Angle class I occlusion. Studies have indicated that the position of the hyoid bone shifts forward following the advancement of the mandible position as a result of orthognathic surgery. The hyoid bone similarly shifts backward after mandibular setback surgery [6–9].

The operation of mandibular setback also simultaneously moved the tongue backward. The tongue root is the back part of the tongue. It connects to the hyoid bone by the* hyoglossal muscle *and* genioglossal muscle,* to the soft palate by the glossopalatine arch, and to the pharynx by the superior pharyngeal constrictor muscle [1–3]. Yamaoka et al. [10] observed that the tongue roots of patients with Angle class II occlusion were farther backward than were those of the patients with Angle class III occlusion.

Therefore, in this study, we investigated whether the action of the hyoid bone and tongue on the mandible affects the stability of the mandible position following intraoral vertical ramus osteotomy (IVRO) treatment for mandibular prognathism. The null hypothesis was that postoperative hyoid bone and tongue changes would influence the postsurgical stability of the mandible position.

## 2. Materials and Methods

### 2.1. Patient Selection

According to power calculations, a sample size of 35 participants would assure a power = .80 at a significance level (alpha) of .05. Normality test (*p* > 0.05) concluded that the data came from a normal distribution. Therefore, 37 patients (26 women and 11 men with a mean age of 20.8 years) from the Division of Oral Maxillofacial Surgery at Kaohsiung Medical University Chung-Ho Memorial Hospital who fulfilled the following criteria were included in the study. The exclusion criteria were as follows: patients with (1) maxillofacial trauma and (2) congenital craniofacial anomalies. The inclusion criteria for the participants of this study were as follows: (1) patients with mandibular prognathism; (2) patients whose mandibular growth and development had already ceased; (3) patients who were followed up regularly in the first year with serial X-ray (T1: before surgery, T2: immediately after surgery, T3: between six weeks and three months after surgery, and T4: more than one year after surgery). All patients were operated on by only one surgeon using IVRO without a genioplasty.

### 2.2. Study Design

**Figure 1:**
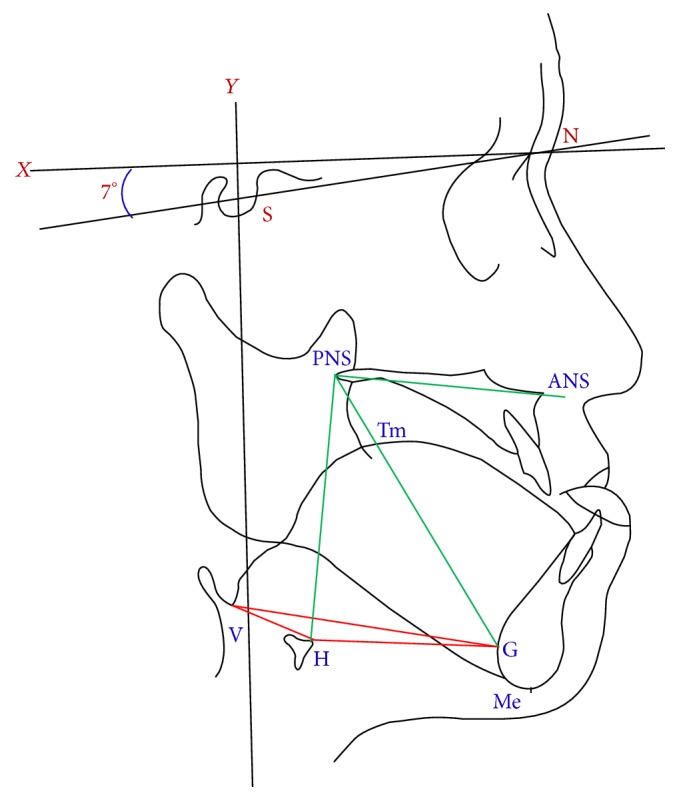
Reference lines and landmarks used in the lateral cephalometric analysis. S: sella. N: nasion. ANS: anterior nasal spine. PNS: posterior nasal spine. Me: the most inferior point on the mandibular symphysis. G: the most prominent point of the mandibular symphyseal posterior border. H: hyoid bone, the most superior and anterior point of hyoid bone. V: vallecula epiglottica. Tm: midpoint of tongue dorsum, the point of tongue intersecting by G-PNS line. *x*-axis: constructed by drawing a line through nasion 7° up from SN line. *y*-axis: constructed by drawing a line through sella (S) perpendicular to the *X*-axis. H-G: distance between H and G. H-V: distance between H and V. Tongue depth: distance between Tm and G. Tongue base length: distance between V and G. G-PNS-ANS angle. H-PNS-ANS angle. G-H-V angle.

Reference points (Figure 1) were including S (sella), N (nasion), ANS (anterior nasal spine), PNS (posterior nasal spine), Me (menton: the most inferior point on the mandibular symphysis), G (the most prominent point of the mandibular symphyseal posterior border), H (most superior and anterior point of hyoid bone), V (vallecula epiglottica), and Tm (midpoint of tongue dorsum: the point of tongue intersecting by G-PNS line). Reference lines were *X*-axis (constructed by drawing a line through nasion 7° up from SN line) and *Y*-axis (constructed by drawing a line through S point perpendicular to the *X*-axis). Linear distances were measured including H-G (distance between H and G), H-V (distance between H and V), tongue depth (distance between Tm and G), and tongue base length (distance between V and G). Angular degrees were investigated including G-PNS-ANS, H-PNS-ANS, and G-H-V. The surgical changes were defined as follows: postsurgical immediate change (T2-T1), difference between postsurgical immediate change and 6 weeks to 3 months' change (T3-T2), change between postsurgical 6 weeks to 3 months and over 1 year (T4-T3), over 1 year final surgical change (T4-T1), and over 1 year surgical stability (T4-T2). These cephalometric landmarks were manually superimposed and identified twice by author (*Chun-Ming Chen*). Intrainvestigator reliability was good (correlation coefficient: 0.997, *p* < 0.001).

### 2.3. Statistical Analysis

IBM®SPSS Statistics 20 was applied for statistical analysis and a *p* value < 0.05 was considered significant. The postoperative changes in the landmarks during each period were identified for statistical analyses comprising mean values, standard deviations, and paired *t*-test. We also used the Pearson correlation coefficient to analyse the statistical significance of changes in mandible position and hyoid bone position. Multiple linear regression analysis was conducted to clarify the factors contributing to postoperative relapse. This retrospective case study followed the principles of the Declaration of Helsinki and was approved by the human investigation review committee at the Kaohsiung Medical University Hospital (*KMUH-IRB-20140173*).

## 3. Results

With regard to presurgery and immediately postsurgery differences (T2-T1) in horizontal direction (Table 1), five related landmarks moved significantly backward (Me: 12.8 mm; G: 13 mm; H: 4.9 mm; Tm: 4.6 mm; V: 5.8 mm). In the vertical direction (Table 2), three related landmarks moved significantly downward (G: 1.9 mm; H: 10.7 mm; V: 6 mm) and Tm moved significantly upward by 2.3 mm. The distances of H-G and tongue base length decreased significantly by 6.5 mm and 5.4 mm, respectively (Table 3). In contrast, H-V distance increased significantly by 2.3 mm. The G-PNS-ANS and H-PNS-ANS angles increased significantly by 6.8° and 2.6°, respectively (Table 4). G-H-V angle decreased significantly by 28.4°. Immediate postsurgery and three-month postsurgery differences (T3-T2) were as follows. Me and G were still 3.3 mm and 2.8 mm backward, and this was statistically significant. Although H also moved 1.5 mm backward, this was not statistically significant. In the vertical direction, H and V moved significantly upward by 8.8 mm and 5.1 mm, respectively. The measurements of all four linear distances decreased significantly. G-H-V angle increased significantly by 23.9°.

Regarding differences between three months (T3) and one year aftersurgery (T4), Me and G moved significantly forward by 3.1 mm and 2.7 mm, respectively, and Tm moved significantly upwards by 2.4 mm. Tongue depth increased by 2.6 mm, which was statistically significant. The G-PNS-ANS and H-PNS-ANS angles narrowed by 2.2° and 1.6°, respectively, and these differences were statistically significant. The differences between presurgery (T1) and one year after surgery (T4) were as follows. Me, H, G, Tm, and V setback were 12.9, 4.8, 13.1, 5, and 4.9 mm, respectively. These differences were all statistically significant. In the vertical direction, Tm moved upward by 4 mm and this change was significant. H-G distance and tongue base length decreased significantly by 7.6 mm and 7.4 mm, respectively. G-PNS-ANS and H-PNS-ANS angles increased significantly by 6.1° and 2.3°, respectively. The G-H-V angle decreased significantly by 6.8°. Investigating postoperative stability (T4-T2), the horizontal changes in Me and point H were very stable with no statistical significance. But, in the vertical direction, points H, G, and V all exhibited statistically significant upward movement (9 mm, 3.1 mm, and 5.1 mm, resp.). The H-V distance decreased significantly by 2 mm. The G-H-V angle increased significantly by 21.6°.

We used Pearson's analysis (Table 5) to test the horizontal and vertical differences in Me as well as hyoid bone and tongue position changes between the period immediately after surgery and one year after surgery (T4-T2) to examine the correlation between these factors. Horizontal changes in Me one year after surgery (T4-T2) were associated only with the amount of setback (correlation coefficient (*r*) = −0.512, *p* < 0.01). Vertical changes in Me one year after surgery were associated with the amount of setback (*r* = −0.347, *p* < 0.05) as well as postsurgery vertical changes in Me (*r* = −0.586, *p* < 0.01). The stability of point Me on the mandible one year after surgery did not correlate with hyoid bone or tongue position change. Multiple linear regression model (*R* ^2^ = 0.2658; *p* < 0.05) is as follows: Horizontal Relapse Me* (T4-T2) = −6.406 − 0.488Me (T2-T1) + 0.069H (T2-T1) − 0.0619V (T2-T1)*. It represented good prediction for postoperative horizontal relapse* (T4-T2)*.

## 4. Discussion

After surgical correction of mandibular prognathism, patients and surgeons focus most on the stability of the mandible. If the mandible is unstable after surgery, occlusion is also unstable, and mandibular protrusion may recur. Majority of the studies on postoperative mandibular stability have focused on the mandible itself. These studies have addressed the following topics: (1) the relationship between various surgical procedures and postoperative stability [11, 12]; (2) the relationship between the amount of setback and stability [13]; (3) methods for postoperative intermaxillary fixation [11, 14]; (4) methods for intersegment fixation [14]; (5) the position and rotation of the condylar segment [15]; (6) the influence of osteotomy position [16]; and (7) the influence of alteration in the gonial angle [17].

The mean mandibular setback in our patients immediately after surgery was larger than the data reported in other studies. Moreover, from immediately to 3 months postoperatively (T3-T2), the mandible continued to show significant backward movement. This may be related to the fact that the IVRO technique does not involve any fixation between the proximal and distal bone segments. Despite six weeks of maxillomandibular fixation, the proximal and distal bone segments had not completely healed. In addition, the condyle had moved downward immediately after surgery, and a new relationship had not been fully established between the condyle and fossa. Together with the action of gravitation, these factors all contributed to further backward movement of the mandible in the six-week to three-month postsurgery period (T3) compared with the period immediately after surgery (T2). Further observation showed that the mandible moved forward significantly after three months after surgery, and its position tended to be stable more than one year postoperatively. In other words, there was no significant change in the position of mandible from immediately after surgery to over one year postoperatively. The vertical changes in mandible position were not significant in any period; that is, the mandible had a stable and very small amount of superoinferior change in the vertical direction.

Investigating the immediate postoperative changes, Hasebe et al. [18] observed pogonion (Pog) setback of 8.4 mm and hyoid bone (H) setback of 4.9 mm, indicating an H/Pog ratio of 58.3% and a hyoid bone downward movement of 7.5 mm. Eggensperger et al. [19] reported menton (Me) setback of 6.3 mm and hyoid bone setback of 2.5 mm, indicating an H/Me ratio of 39.7% and a hyoid bone downward movement of 7.5 mm. In our study, menton setback was 12.8 mm and hyoid bone setback was 4.9 mm, indicating an H/Me ratio of 38.3% and a significant downward movement of 10.7 mm. These showed that mandibular setback did not entirely influence hyoid bone position. These phenomena are accompanied by the antagonist of suprahyoid and infrahyoid muscles.

Investigating the distance between the hyoid and mandible, Hwang et al. [20] found that H-G length increased by 1.85 mm immediately after surgery. However, we observed that H-G length decreased by 6.5 mm (−6.5/45.5 = −14.3%) immediately after surgery. Our report revealed converse results and this may be due to the smaller amount of setback in Hwang et al.'s study. Therefore, the flexibility of H-G length can endure a certain amount of mandibular setback. With regard to the distance between the hyoid bone and the vallecula epiglottica (V: root of tongue), H-V length increased significantly immediately after surgery because V setback was greater than H setback. Moreover, the increase in the G-PNS-ANS angle was significantly higher than the increase in the H-PNS-ANS angle, indicating that G setback was greater than H setback. The G-H-V angle narrowed significantly after surgery, by 28.4°; this was the result of the significant downward movement (10.7 mm) of H.

Six weeks after surgery (T3), the hyoid bone continued to move backward and then shifted forward three months after surgery. However, these changes were not significant, indicating that the coordination between the suprahyoid and the infrahyoid muscles affected the postoperative stability of the hyoid bone more than the changes in the mandible. The suprahyoid and infrahyoid muscles drag the hyoid bone back to near its original position. Thus, the hyoid bone moved significantly upward by 8.8 mm, causing the G-H-V angle to increase significantly by 23.9°. During mandibular setback, the whole tongue also extruded toward the rear. However, the pull of the hyoid bone and the necessity to maintain sufficient pharyngeal airway space may have affected this phenomenon. As the vallecula epiglottica is located next to the hyoid bone, changes in the position of the hyoid bone substantially influence the vallecula epiglottica.

At one year after surgery, Gu et al. [21] observed a Pog setback of 9.7 mm and a hyoid bone setback of 4.88 mm, indicating a H/Pog ratio of 50.3% and a hyoid bone downward movement of 1.72 mm; Eggensperger et al. [19] noted an Me setback of 5.4 mm and a hyoid bone setback of 2 mm, indicating an H/Me ratio of 37% and a hyoid bone downward movement of 3.5 mm. In our study, at one year after surgery, Me setback was 12.9 mm and H setback was 4.8 mm, indicating an H/Me ratio of 37.2% and a hyoid bone downward movement of 1.7 mm. As a consequence of the new mandible position, the G-H-V angle decreased significantly (6.8°); this was consistent with the postoperative hyoid bone downward position and this represents an adaptation of anatomical organization.

On the basis of these results, the aforementioned studies have indicated that the ratio of hyoid/mandible setback immediately after surgery and one year after surgery was stable, at approximately 40–50%. Hwang et al. [20] reported that H-G length had decreased by 3.96 mm at 1.5 years after surgery. Eggensperger et al. [19] reported Me setback of 6.3 mm and a 1 mm decrease in H-G length (−1/40.1 = −2.4%) one year after surgery. In the present study, we observed an H-G length decrease of 7.6 mm (−7.6/45.5 = −16.7%) one year after surgery. The reduction ratio was the highest in the present study, possibly because our patients experienced the highest amount of setback.

As a result of mild tongue elevation immediately after surgery, tongue depth decreased by 0.8 mm and this was not significant. Because of compression by mandibular setback, tongue base length decreased significantly by 5.4 mm. Six weeks to three months postoperatively (T3), the mandible was still significantly backward along with a significantly decreased tongue depth and tongue base length. Three months to over one year after surgery (T4-T3), the mandible was significantly forward along with a significantly increased tongue depth. However, the vallecula epiglottica had also moved forward and tongue base length had increased, but not significantly. Our study showed significantly decreased tongue base length but no change in tongue depth, more than one year after surgery (T4-T1).

Because mandibular setback simultaneously moved the hyoid bone and tongue backward, we used the hyoid-related position and linear and angular measurements as variables to investigate postoperative mandibular stability. After Pearson correlation analysis, changes in linear distances, including H-G, H-V, tongue depth, and tongue base length, did not significantly influence postoperative mandibular stability. Postsurgery changes in angular measurements, including G-PNS-ANS, H-PNS-ANS, and G-H-V angles, also did not significantly influence postoperative mandibular stability. Therefore, the null hypothesis was rejected. Therefore, we concluded that postoperative hyoid bone and tongue changes did not influence the postsurgical stability of the mandible. Our finding was different to Gu et al.'s report [21] which suggested that the forward relapse of Pog correlated with the change of hyoid position three years after surgery.

In our study, horizontal stability of point Me on the mandible one year after surgery did not correlate with hyoid bone or tongue position change. Therefore, the null hypothesis was rejected. However, horizontal stability correlated significantly with the amount of mandibular setback (*r* = −0.512, *p* < 0.01) immediately after surgery. Our finding is similar to previous studies that concluded that the amount of surgical setback correlated significantly with postoperative mandibular relapse. Moreover, vertical stability correlated significantly with both the amount of mandibular setback in the horizontal (*r* = −0.347, *p* < 0.05) and the vertical (*r* = −0.586, *p* < 0.01) directions. Multiple linear regression model represented good prediction for postoperative horizontal relapse* (T4-T2)*.

## 5. Conclusion

Changes of hyoid, tongue, and their related dimensions presented no correlation with postoperative mandibular relapse. However, postoperative stability was correlated significantly with the amount of mandibular setback. In other words, greater mandibular setback was associated with greater postoperative relapse.

## Figures and Tables

**Table 1:** Values for the various cephalometric parameters of the serial postsurgical changes in the horizontal direction.

Variable (mm)	T2-T1			T3-T2			T4-T3			T4-T1			T4-T2		
	Mean	SD		Mean	SD		Mean	SD		Mean	SD		Mean	SD	
Me	−12.8	4.60	*∗*	−3.3	5.29	*∗*	3.1	5.44	*∗*	−12.9	4.36	*∗*	−0.1	4.20	—
G	−13.0	6.68	*∗*	−2.8	4.64	*∗*	2.7	4.81	*∗*	−13.1	6.11	*∗*	−0.1	3.87	—
H	−4.9	7.30	*∗*	−1.5	7.61	—	1.7	7.23	—	−4.8	5.32	*∗*	0.2	6.27	—
Tm	−4.6	3.86	*∗*	0.6	3.34	—	−1.0	3.50	—	−5.0	4.44	*∗*	−0.4	2.84	—
V	−5.8	7.07	*∗*	0.4	6.39	—	0.4	6.15	—	−4.9	4.59	*∗*	0.8	6.69	—

(+) means the forward movement; (−) means the backward movement.

*∗*: significant, *p* < 0.05.

—: not significant.

T2-T1: postsurgical immediate change; T3-T2: difference between postsurgical immediate change and 6 weeks to 3 months' change.

T4-T3: change between postsurgical 6 weeks to 3 months and over 1 year.

T4-T1: over 1 year final surgical change; T4-T2: over 1 year surgical stability.

**Table 2:** Values for the various cephalometric parameters of the serial postsurgical changes in the vertical direction.

Variable (mm)	T2-T1			T3-T2			T4-T3			T4-T1			T4-T2		
	Mean	SD		Mean	SD		Mean	SD		Mean	SD		Mean	SD	
Me	1.6	5.24	—	−0.4	3.82	—	−1.5	7.33	—	−0.3	5.62	—	−1.9	6.75	—
G	1.9	3.94	*∗*	−1.9	4.31	*∗*	−1.2	7.27	—	−1.2	5.48	—	−3.1	5.87	*∗*
H	10.7	5.79	*∗*	−8.8	4.48	*∗*	−0.2	8.84	—	1.7	7.97	—	−9.0	8.52	*∗*
Tm	−2.3	4.67	*∗*	0.7	5.30	—	−2.4	6.77	*∗*	−4.0	5.90	*∗*	−1.7	5.69	—
V	6.0	5.32	*∗*	−5.1	5.36	*∗*	0.0	8.19	—	0.9	7.01	—	−5.1	7.50	*∗*

(+) means the downward movement; (−) means the upward movement.

*∗*: significant, *p* < 0.05.

—: not significant.

T2-T1: postsurgical immediate change; T3-T2: difference between postsurgical immediate change and 6 weeks to 3 months' change.

T4-T3: change between postsurgical 6 weeks to 3 months and over 1 year.

T4-T1: over 1 year final surgical change; T4-T2: over 1 year surgical stability.

**Table 3:** Linear distances (mm) for the various cephalometric parameters of the serial postsurgical changes.

Linear distance	T2-T1			T3-T2			T4-T3			T4-T1			T4-T2		
	Mean	SD		Mean	SD		Mean	SD		Mean	SD		Mean	SD	
H-G	−6.5	5.76	*∗*	−2.4	4.83	*∗*	1.3	4.28	—	−7.6	5.49	*∗*	−1.1	5.34	—
H-V	2.3	4.79	*∗*	−1.8	4.36	*∗*	−0.2	2.94	—	0.3	3.22	—	−2.0	4.02	*∗*
Tongue depth	−0.8	6.30	—	−3.2	7.22	*∗*	2.6	6.12	*∗*	−1.5	5.58	—	−0.6	5.21	—
Tongue base	−5.4	5.42	*∗*	−2.7	5.37	*∗*	0.7	4.70	—	−7.4	6.28	*∗*	−2.0	5.98	—

*∗*: significant, *p* < 0.05.

—: not significant.

T2-T1: postsurgical immediate change; T3-T2: difference between postsurgical immediate change and 6 weeks to 3 months' change.

T4-T3: change between postsurgical 6 weeks to 3 months and over 1 year.

T4-T1: over 1 year final surgical change; T4-T2: over 1 year surgical stability.

**Table 4:** Angular measurements for the various cephalometric parameters of the serial postsurgical changes.

Angular degrees (°)	T2-T1			T3-T2			T4-T3			T4-T1			T4-T2		
	Mean	SD		Mean	SD		Mean	SD		Mean	SD		Mean	SD	
G-PNS-ANS	6.8	4.23	*∗*	1.6	3.55	*∗*	−2.2	3.35	*∗*	6.1	4.71	*∗*	−0.6	3.39	—
H-PNS-ANS	2.6	5.55	*∗*	1.3	4.18	—	−1.6	4.56	*∗*	2.3	5.47	*∗*	−0.3	4.83	—
G-H-V	−28.4	18.88	*∗*	23.9	18.82	*∗*	−2.3	18.60	—	−6.8	17.23	*∗*	21.6	18.69	*∗*

*∗*: significant, *p* < 0.05.

—: not significant.

T2-T1: postsurgical immediate change; T3-T2: difference between postsurgical immediate change and 6 weeks to 3 months' change.

T4-T3: change between postsurgical 6 weeks to 3 months and over 1 year.

T4-T1: over 1 year final surgical change; T4-T2: over 1 year surgical stability.

**Table 5:** Pearson correlation between surgical relapse (T4-T2) and immediate change (T2-T1) in the various parameters.

	Me (T4-T2)			
	Horizontal		Vertical	
	CC		CC	
Landmarks				
Horizontal (mm)				
Me (T2-T1)	−0.512	*∗*	−0.347	*∗*
H (T2-T1)	−0.271	—	−0.199	—
G (T2-T1)	−0.169	—	−0.133	—
Tm (T2-T1)	−0.002	—	−0.139	—
V (T2-T1)	−0.234	—	−0.169	—
Vertical (mm)				
Me (T2-T1)	−0.181	—	−0.586	*∗*
H (T2-T1)	−0.224	—	−0.231	—
G (T2-T1)	0.123	—	−0.218	—
Tm (T2-T1)	0.045	—	−0.045	—
V (T2-T1)	−0.228	—	−0.153	—
Linear distance (mm)				
H-G	0.037	—	−0.046	—
H-V	0.014	—	0.021	—
Tongue depth	−0.111	—	−0.301	—
Tongue base	0.155	—	0.003	—
Angular degrees (°)				
G-PNS-ANS	0.198	—	0.047	—
H-PNS-ANS	0.323	—	0.076	—
G-H-V	0.094	—	0.278	—

CC: correlation coefficients.

*∗*: significant, *p* < 0.05; —: not significant.

T2-T1: postsurgical immediate change.

T4-T2: over 1 year surgical relapse.
